# Silencing of maternal hepatic glucocorticoid receptor is essential for normal fetal development in mice

**DOI:** 10.1038/s42003-019-0344-3

**Published:** 2019-03-15

**Authors:** Matthew A. Quinn, Amy McCalla, Bo He, Xiaojiang Xu, John A. Cidlowski

**Affiliations:** 1 Department of Pathology, Section on Comparative Medicine, Wake Forest School of Medicine, Winston-Salem, North Carolina 27517 USA; 2 Signal Transduction Laboratory, Research Triangle Park, North Carolina USA; 3 Laboratory of Integrative Bioinformatics, National Institute of Environmental Health Sciences, National Institutes of Health, Department of Health and Human Services, Research Triangle Park, North Carolina 27709 USA

## Abstract

Excessive or chronic stress can lead to a variety of diseases due to aberrant activation of the glucocorticoid receptor (GR), a ligand activated transcription factor. Pregnancy represents a particular window of sensitivity in which excessive stress can have adverse outcomes, particularly on the developing fetus. Here we show maternal hepatic stress hormone responsiveness is diminished via epigenetic silencing of the glucocorticoid receptor during pregnancy. Provocatively, reinstallation of GR to hepatocytes during pregnancy by adeno-associated viral transduction dysregulates genes involved in proliferation, resulting in impaired pregnancy-induced hepatomegaly. Disruption of the maternal hepatic adaptation to pregnancy results in in utero growth restriction (IUGR). These data demonstrate pregnancy antagonizes the liver-specific effects of stress hormone signaling in the maternal compartment to ultimately support the healthy development of embryos.

## Abstract (web summary)

Matthew Quinn et al showed diminished hepatic stress responses in pregnant mice due to epigenetic-mediated decreases in glucocorticoid receptor expression. This decrease is necessary for fetal development and highlights the importance of understanding the tissue-specific effects of glucocorticoid activation in models of maternal stress.

## Introduction

Glucocorticoids are steroid hormones that are synthesized and secreted under coordinated actions of the hypothalamic–pituitary–adrenal axis that gets activated in response to a variety of stressful stimuli. Once in the circulation, glucocorticoids regulate a host of physiological processes ranging from immune regulation to altering systemic metabolism^1^. The physiological actions elicited by glucocorticoids are mediated by binding to and activating the glucocorticoid receptor (GR), a ligand-activated transcription factor. While glucocorticoids are essential to return the body to homeostasis following either a physiological or psychological stress, excessive glucocorticoid production or chronic stress is linked to an array of diseases, particularly metabolic diseases and obesity^2–6^.

The biological response to pregnancy is systemic with almost every organ of the body adapting, with the primary goal to support the developing fetus. The liver for example, a mitotically dormant organ in adults, reenters the cell cycle to elicit a pregnancy-induced growth response^7–10^. It is well established that the environment in which the fetus develops can have a long-lasting impact on the health of the offspring post parturition. This is known as the developmental origins of the health and disease hypothesis^11^. While a variety of factors contribute to this phenomenon, maternal stress has been identified as key in driving fetal programming eliciting poor health outcomes in the offspring ranging from neuropsychiatric disease^12–17^ to cardiovascular disease^18–21^ and metabolic syndrome^22–28^. The effects of maternal stress have been attributed to aberrant activation of the glucocorticoid receptor (GR)^29–34^. The physiological mechanisms in which mothers combat maternal stress to avoid these deleterious effects of pathogenic GR signaling during pregnancy have yet to be fully realized.

Previous studies have established an antagonistic relationship between female sex hormones and stress hormone responsiveness in a variety of reproductive tissues^35–40^. Furthermore, we have recently demonstrated that loss of reproductive axis activity through ovariectomy promotes glucocorticoid hypersensitivity and a Cushing-like syndrome resulting in the development of obesity, hyperglycemia, and hepatic steatosis^41^, extending this antagonistic relationship beyond the reproductive tract into metabolic tissues. These findings lay a potential physiological foundation for reproductive axis activation in females, limiting the stress response to avoid deleterious effects of stress during pregnancy.

The effects of GR activation during pregnancy in a tissue-specific fashion have largely been overlooked, as models of maternal stress rely upon systemic administration of glucocorticoids^29–34^. This has led to a lack of understanding of how the maternal compartment regulates the stress response in various tissues and how these tissues contribute to the maladaptive response to stress in utero. Here, we show that during normal pregnancy, hepatic stress hormone responsiveness is dramatically reduced in mice. The decreased glucocorticoid sensitivity seen during pregnancy is a product of reduced GR expression elicited by epigenetic silencing. Furthermore, we found that downregulation of maternal hepatic GR expression is essential to allow for pregnancy-induced liver growth. This was evidenced by the finding that GR reinstallation specifically to hepatocytes blunts pregnancy-induced hepatomegaly resulting in smaller livers. Last and unexpectedly, maternal hepatic GR reinstallation resulted in in utero growth restriction of the fetuses. Our findings have uncovered a role of GR silencing in the maternal liver to allow for the physiological hepatic response to pregnancy and thus appropriate fetal development. The data presented herein demonstrate how aberrant hepatic glucocorticoid signaling can facilitate intrauterine growth restriction (IUGR). Further investigation into how the stress hormone response is regulated in other tissue systems during pregnancy is essential in understanding how maternal stress interferes with the systemic adaptation to pregnancy.

## Results

### Hepatic glucocorticoid desensitization during pregnancy

**Fig. 1:**
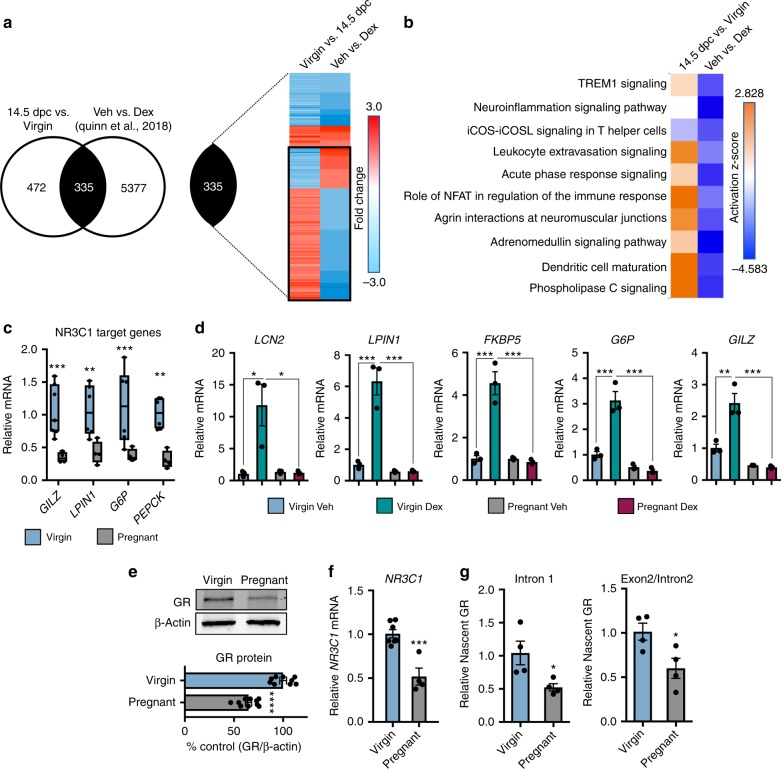
Pregnancy promotes hepatic glucocorticoid resistance via receptor downregulation. **a** Whole-transcriptome array of livers from virgins and mice pregnant at 14.5 dpc overlaid with previously published RNA-seq of glucocorticoid-responsive genes in the virgin female liver (GSE99309). **b** Top physiological pathways activated in the pregnant liver compared with glucocorticoid regulation of those pathways. **c** mRNA expression of GR target genes in pregnant liver. *n* = 4–7 mice per group; df = 33. **d** Real-time PCR analysis of livers from dexamethasone challenge (10 μg/kg) in female virgins and pregnant mice. *n* = 3 mice per group; df = 11. **e** Immunoblot and quantification of GR protein in livers from virgin and pregnant mice. β-actin was used as a loading control and for normalization. *n* = 9–10 animals per group; df = 17. **f** *NR3C1* mRNA in virgin and pregnant livers. *n* = 4–7 mice per group; df = 9. **g** Expression of nascent *NR3C1* mRNAs from virgin and pregnant livers. *n* = 4 mice per group; df = 6. Data are expressed as mean ± SEM. * denotes *p* *<* 0.05, **p < 0.01, ***p < 0.001, ****p < 0.0001 based on a two-sided *T* test when comparing two groups and one-way ANOVA when comparing three or more groups

The liver is well recognized to respond physiologically to pregnancy; however, the transcriptional response underlying these physiological changes is largely unknown. To address this, we performed whole-transcriptome analysis of livers from virgins and pregnant mice at 14.5 dpc via microarray analysis to characterize the transcriptional changes in the liver associated with pregnancy. The transcriptome array revealed that pregnancy alters the expression of ~800 genes in the maternal liver at this time point (Fig. 1a). Comparing the pregnancy-regulated transcriptome with previously published RNA-seq of the hepatic glucocorticoid transcriptome in female mice^41^ (GSE99309) uncovered ~40% (335 genes) of the hepatic pregnancy-regulated genes overlaps with the stress hormone transcriptome in virgin females. Assessing the fold change elicited by pregnancy to the fold change elicited by stress hormone administration in virgin females revealed nearly 70% of commonly regulated genes to be altered in an opposite direction (black box) (Fig. 1a). Furthermore, the top physiological pathways activated during pregnancy are all repressed in the livers of virgin mice treated with glucocorticoids (Fig. 1b), suggesting that the maternal liver diminishes the endogenous stress hormone pathway to achieve the appropriate maternal gene expression profile. In support of this notion, we observed a lower expression of endogenous GR target genes in the liver in response to pregnancy (Fig. 1c). Last, pregnant mice were refractory to inducing GR target genes following the administration of the synthetic GR ligand dexamethasone (Fig. 1d), defining a state of glucocorticoid resistance in the maternal liver.

Glucocorticoid sensitivity can be modulated via multiple mechanisms, particularly by altering glucocorticoid receptor expression^42^. We hypothesized that pregnancy-induced hepatic glucocorticoid resistance is due to the reduction in receptor expression. To support this idea, we observed a decrease in both hepatic GR protein and mRNA in pregnant mice compared with virgin mice (Fig. 1e, f). The decrease in hepatic GR expression during pregnancy is attributed to reduced transcriptional activation, as evidenced by a decrease in the expression of nascent *NR3C1* (gene name for the glucocorticoid receptor) transcripts in pregnant mice (Fig. 1g). We propose that a reduced *NR3C1* expression is due to epigenetic silencing, potentially through altered promoter methylation and subsequent repressor recruitment (Supplementary Figure 1).

### GR reinstallation impairs pregnancy-induced hepatomegaly

**Fig. 2:**
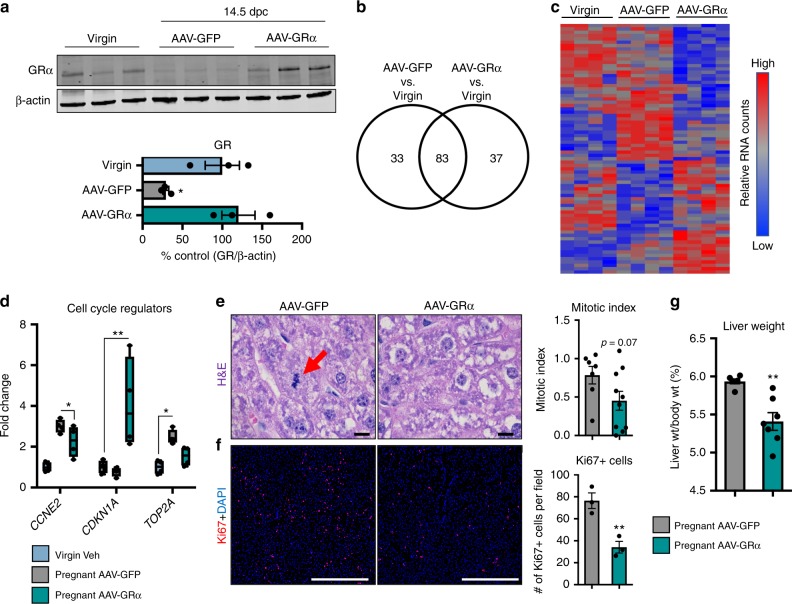
GR reinstallation in the liver disrupts pregnancy-induced hepatomegaly. **a** Immunoblot of GR in livers of virgin and pregnant AAV-GFP- and AAV-GR-injected mice and quantification. *n* = 3 mice per group; df = 8. **b** Venn diagram of differentially expressed genes assessed by Nanostring (*n* = 4 mice per group) in response to pregnancy in AAV-GFP and AAV-GR mice. **c** Heatmap of differentially expressed genes from Nanostring analysis. *n* = 4 mice per group. **d** Fold change of *CCNE2*, *CDKN1a*, and *TOP2a* in AAV-GFP and AAV-GR mice normalized to virgins. *n* = 4 mice per group; df = 27. **e** H&E staining of livers from pregnant AAV-GFP and AAV-GR mice. The red arrow indicates a mitotic figure. Mitotic index of livers from pregnant AAV-GFP and AAV-GR mice. *n* = 7–10 mice per group; df = 15. Scale bar is 1000 μm**. f** Immunofluorescence for Ki67 from livers of pregnant AAV-GFP and AAV-GR mice. Quantification of Ki67 + nuclei per field. *n* = 3 mice per group; df = 4. Scale bar is 100 μm. **g** Liver to body weight percent in pregnant AAV-GFP and AAV-GR mice. *n* = 6–7 mice per group; df = 11. * denotes *p* < 0.05, **p < 0.01, ***p < 0.001 based on a two-sided *T* test when comparing two groups and one-way ANOVA when comparing three or more groups

To determine the relevance and importance of suppression of hepatic GR signaling during pregnancy, we reinstalled GR expression specifically to hepatocytes via adeno-associated viral transduction with a GR vector (AAV-GR) under the human alpha-1 antitrypsin promoter as previously described^43^. The AAV-GR mice were compared with their age- and gestational-matched counterparts transduced with a control vector (AAV-GFP) (Fig. 2a). Transcriptional profile data of livers from pregnant mice within our studies and other reports suggest a pro-cancer or oncogenic-like gene signature in the liver during pregnancy that promotes proliferation^7^. The functional necessity of the pregnancy-induced pro-cancer gene expression profile is to induce otherwise mitotically dormant hepatocytes^8–10^ to reenter the cell cycle and meet the increasing metabolic demands incurred during pregnancy. To evaluate if hepatic GR activation disrupts pregnancy-regulated genes involved in growth and proliferation, we performed Nanostring analysis on livers from virgins and pregnant AAV-GFP mice and pregnant AAV-GR and surveyed genes involved in cell-cycle regulation and proliferation. We found that reinstallation of hepatic GR during pregnancy alters the pro-cancer gene expression program, leading to gain and loss of function of gene regulation, particularly within those genes involved in cell-cycle regulation (Fig. 2b–d). Our Nanostring data indicate that hepatic GR reinstallation during pregnancy disrupts the innate pregnancy-induced proliferative gene expression profile. The functional consequence of increased GR signaling during pregnancy was observed histologically as a decrease in the proliferative capacity of hepatocytes, as evidenced by a decrease in the presence of mitotic figures as well as reduced Ki67 + nuclei (Fig. 2e, f). The disrupted maternal hepatic growth response ultimately culminates in a reduced liver to body weight ratio in pregnant AAV-GR-injected dams (Fig. 2g). Collectively, our data indicate that maternal hepatic glucocorticoid resistance is likely established to allow pregnancy-induced hepatomegaly to ensue.

### Hepatic GR reinstallation promotes fetal growth restriction

**Fig. 3:**
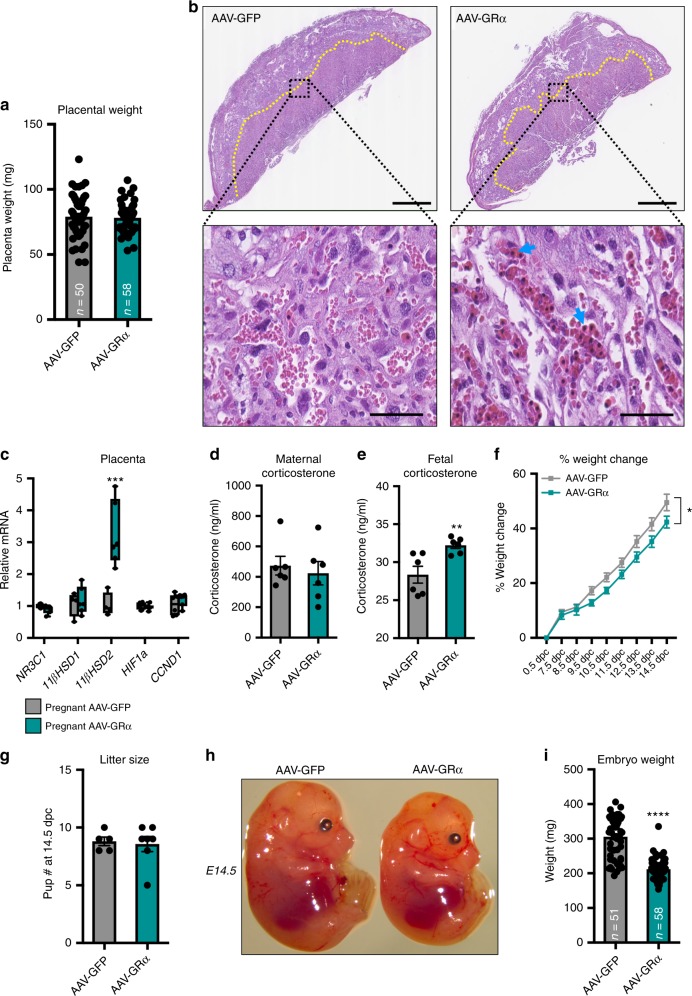
Hepatic GR reinstallation during pregnancy elicits in utero fetal growth restriction. **a** Placenta weight from AAV-GFP- and AAV-GR-injected mice. *n* = 50–58 placentas per group; df = 106. **b** H&E staining of placentas from AAV-GFP and AAV-GR mice at 14.5 dpc. Yellow line marks the zone between the junctional and labyrinth zone. Scale bar is 500 μm. Inset is the zoom-in of the labyrinth zone. Blue arrows indicate nucleated red blood cells. Scale bar is 100 μm. **c** Gene expression of GR signaling and hypoxia genes in placentas of AAV-GFP and AAV-GR mice. *n* = 4–6 placentas per group; df = 46. **d** Circulating corticosterone in pregnant AAV-GFP and AAV-GR mice. *n* = 6 mice per group; df = 10. **e** Fetal corticosterone in pups from AAV-GFP and AAV-GR dams. *n* = 6 pups per group; df = 10. **f** Percentage gain in pregnant AAV-GFP and AAV-GR mice from 0.5 to 14.5 dpc. *n* = 4–7 mice per group; df = 85. **g** In utero litter size at 14.5 dpc in pregnant AAV-GFP and AAV-GR mice. *n* = 5–7 mice per group; df = 10. **h** Gross images of pups from pregnant AAV-GFP and AAV-GR mice at E14.5. **i** Embryo weights of pups from pregnant AAV-GFP and AAV-GR mice at E14.5. *n* = 51–58 pups per group; df = 107. * denotes *p* < 0.05, **p < 0.01, ***p < 0.001, ****p < 0.0001 based on a two-sided *T* test

With data indicating that increased GR expression can adversely affect pregnancy-induced hepatomegaly, we hypothesized that GR-induced hepatic changes in the maternal compartment would have downstream effects on the developing fetuses. Maternal stress is well appreciated to have detrimental effects on placental physiology^44–53^. Therefore, we initially examined if activation of maternal hepatic GR promotes placental dysfunction. Maternal hepatic stress hormone signaling did not elicit overt gross placental changes, as evidenced by comparable placental weights at 14.5 dpc between AAV-GFP- and AAV-GR-injected mice (Fig. 3a). Sub-gross histological examination showed no morphological differences in zonation of placentas between AAV-GFP- and AAV-GR-injected mice (Fig. 3b). At higher magnification (40×) of the labyrinth zone, we observed a marked increase in the presence of nucleated red blood cells in AAV-GR-injected mice (Fig. 3b, blue arrows), a well-recognized sign of fetal stress in utero^54^. To evaluate potential fetal stress, we examined the expression of genes within the placenta involved in stress hormone signaling as well as hypoxia to determine if placental stress underlies the increased nucleated red blood cells in AAV-GR-injected mice. We neither observed overt changes in hypoxia-related genes, placental VEGF, nor stress hormone signaling genes, except for 11β-hydroxysteroid dehydrogenase type II (*HSD11B2*) (Fig. 3c and Supplementary Figure 2). 11β-HSD2 is key in the inactivation of cortisol/corticosterone to its inactive metabolite cortisone/11-dehydrocorticosterone. We speculated that the altered 11β-HSD2 expression is driven by dysregulated maternal corticosterone metabolism. However, no differences in the circulating glucocorticoid levels between AAV-GFP- and AAV-GR-injected pregnant mice were found (Fig. 3d), suggesting that an alternative signaling mechanism underlies the increased placental 11β-HSD2 expression in AAV-GR-injected dams. Alternatively, fetal corticosterone could be elevated, leading to enhanced placental 11β-HSD2 expression as a compensatory mechanism. In line with this, we did observe a modest, but consistent increase in fetal corticosterone levels (Fig. 3e).

The increased nucleated red blood cells in the placenta coupled to increased fetal corticosterone levels prompted us to investigate the effects of maternal hepatic GR reinstallation on the fetuses in utero. Pregnant mice that have hepatic GR reinstalled display a delay in weight gain compared with pregnant controls (Fig. 3f). Of note, the smaller liver sizes in pregnant AAV-GR mice (Fig. 2g) did not account completely for the differences in total body weight between the groups. Litter size was monitored to determine if weight differences were due to a failure to support the same number of embryos in utero. A comparable number of embryos between groups at 14.5 dpc indicates that hepatic GR reinstallation does not lead to a failure to support pregnancy (Fig. 3g). While pup number was the same between groups, the size of pups between AAV-GFP and AAV-GR differed considerably, with pups from AAV-GR dams being smaller (Fig. 3h, i). These data indicate that hepatic GR reinstallation ultimately promotes fetal stress culminating in IUGR.

## Discussion

In the current study, we aimed to determine the effects of pregnancy on maternal hepatic glucocorticoid signaling. First, we found that the pregnancy-regulated hepatic transcriptome shows a large overlap with the glucocorticoid-regulated transcriptome. Of the intersecting transcriptomes, a vast majority of the genes and biological pathways are regulated in the opposite direction. This led us to hypothesize that pregnancy leads to a state of hepatic glucocorticoid insensitivity. To this end, we uncovered that under normal physiological conditions, pregnancy leads to a decrease in hepatic GR expression, which ultimately promotes decreased glucocorticoid responsiveness. By restoring hepatic GR expression during pregnancy with the use of AAV-mediated viral transduction, we uncovered that one of the physiological consequences of decreased maternal hepatic GR signaling is to allow for pregnancy-induced hepatomegaly to ensue. This was evidenced by the fact that GR reinstallation to the liver during pregnancy led to dysregulation of a cohort of genes involved in cell-cycle regulation. The dysregulated gene expression profile ultimately resulted in impaired hepatic proliferation and decreased liver weights in pregnant mice that have hepatic GR reinstalled. Finally, we discovered that hepatic GR downregulation is essential for normal embryonic development. Embryos whose mothers received AAV-GR displayed altered placental expression of 11β-HSD2 and had higher fetal corticosterone levels compared with pregnant AAV-GFP controls. Furthermore, mothers receiving AAV encoding for GR had delayed weight gain during the course of gestation due to decreased embryo weights. In sum, we have uncovered a pathogenic role for hepatic GR signaling in disrupting the hepatic adaptation to pregnancy and ultimately promoting intrauterine fetal growth restriction.

Pregnancy is characterized by enhanced systemic production of glucocorticoids in both humans and rodents^55^. One way the mother compensates for the hyperglucocorticoidism observed during pregnancy is through the increased production of corticosteroid-binding globulin (CBG), a protein which binds circulating glucocorticoids rendering them biologically inactive^56^. While CBG increases at a comparable rate to glucocorticoids during pregnancy, high levels of sex hormones, such as progesterone, can displace glucocorticoid binding to CBG, rendering them biologically active in the circulation^57^. The physiological importance of active stress hormones during pregnancy is highlighted by a recent report by Whirledge et al. showing that uterine-specific GR knockout mice display impaired implantation and decidualization, which ultimately leads to a sub-fertile phenotype^58^. While the uterine effects of glucocorticoids are now appreciated, nothing was known about the effects of stress hormone signaling, specifically in the liver during pregnancy. We found that unlike the uterus, the liver requires silencing of GR for both the maternal adaptation to pregnancy as well as fetal development. We speculate that the physiological downregulation of hepatic GR during pregnancy is a compensatory mechanism to avoid pathogenic glucocorticoid signaling in the liver during the Cushing-like state observed throughout pregnancy. One reason for this is that the liver needs to undergo growth to support the metabolic demand of pregnancy. Due to the increased proliferation in the liver during pregnancy, liver regeneration is actually restored in rodents in response to pregnancy^9^. In rats undergoing partial hepatectomy, glucocorticoid administration inhibits the regenerative process^59^. By downregulating hepatic GR during gestation, pregnancy is able to elicit liver growth and enhance the regenerative process. Further studies aimed at evaluating the regenerative response in the liver during pregnancy in the context of partial hepatectomy following GR reinstallation are needed. Another pathogenic effect of excessive GR activation is the onset of cholestatic liver disease, a disorder of excessive bile acid production^60,61^. While not investigated in the current study, aberrant maternal hepatic glucocorticoid signaling could potentially underlie the idiopathic nature of cholestasis of pregnancy and warrants further investigation.

GR sensitivity can be modulated at several levels. For example, post-translational regulation of GR is a dynamic way by which the glucocorticoid response can be modulated. We have previously demonstrated that in a mouse model of menopause, systemic levels of follicle-stimulating hormone (FSH) can increase hepatic glucocorticoid sensitivity through enhancing ligand-dependent phosphorylation of GR, which ultimately promotes the development of fatty liver disease^41^. Given the sensitizing effects of FSH on hepatic GR activation, it is possible that the low levels of circulating FSH during pregnancy could account, at least in part, to the diminished stress hormone responsiveness in the maternal liver and warrant further investigation. As an alternative, methylation is an epigenetic means by which GR expression can be controlled. We show that hypomethylation of the *NR3C1* CGI shore and CGI and subsequent enhanced YY1 co-repressor recruitment contribute to hepatic GR downregulation in gravid mice (Supplementary Figure 1). Mechanistically, we propose that pregnancy-induced hepatic *NR3C1* hypomethylation is a product of passive dilution of 5mC. This model (Supplementary Figure 1f) is based on the findings that the liver displays global increases in the DNA methyltransferase substrate *S-adnosylmethionine* (SAM) (Supplementary Figure 1a, also refer to Supplementary Methods). We hypothesize that the increased hepatic SAM levels observed during pregnancy are due to decreased DNA methyltransferase substrate utilization. Supporting this hypothesis, we observe a decreased global hepatic 5mC in the pregnant mouse liver compared with virgins (Supplementary Figure 1b). Last, we discovered that *DNMT1*, the maintenance DNA methyltransferase responsible for methylating a newly synthesized DNA, is selectively downregulated in the liver during pregnancy, while the de novo methyltransferases *DNMT3a* and *DNMT3b* are unaffected (Supplementary Figure 1c). The enhanced proliferative capacity observed in the liver during pregnancy coupled to decreased *DNMT1* expression and the subsequent decreased total 5mC leads us to propose a model, in which pregnancy promotes passive dilution of DNA methylation of the *NR3C1* promoter and potential YY1 repressor recruitment and epigenetic silencing (Supplementary Figure 1d–f). While the current study only surveyed the *NR3C1* methylation landscape, it is possible that other loci in other tissues are subjected to hypomethylation as a means to regulate gene expression during pregnancy. In fact, it has recently been shown in blood samples that pregnancy elicits a decrease in global DNA methylation^62^. Moreover, dysregulation of maternal DNA methylation is linked to gestational diabetes^63^. We believe that the dynamic nature of maternal DNA methylation patterns and the methyl-sensitive transcriptional regulators in various tissues and at different loci is of great importance to understand and to gain insight into the physiology and pathophysiology of pregnancy.

Maternal stress is a well-established factor promoting fetal growth restriction. The classical role of maternal stress that elicits intrauterine growth restriction is through the alteration of placental function. For example, excessive maternal glucocorticoids can impair trophoblast function, leading to decreased proliferation, migration, and invasion, ultimately culminating in pre-eclampsia-like symptoms in rats^49^. Furthermore, in a sheep model of maternal stress, glucocorticoid administration leads to cell death of the placenta^50^. Our results extend beyond the attributed role of stress hormones in decreasing placental function to promote intrauterine growth restriction and show that hepatic stress hormone signaling can also have detrimental effects in the onset of fetal growth restriction. Further studies focused on the long-lasting effects of our model of fetal growth restriction post parturition are essential, as in utero growth restriction has been shown to lead to diseases in the offspring extending into adulthood^64^.

Despite a long-standing relationship between maternal stress and detrimental fetal outcomes, the molecular mechanisms and tissue-specific effects elicited by stress during pregnancy have largely remained elusive. Our present study in conjunction with previous reports reveal a precarious role of stress hormone signaling in different tissues during pregnancy in the regulation of embryo development. Understanding the tissues that require GR activation versus tissues with which glucocorticoid signaling needs to be silenced will be instrumental in uncovering the physiological and pathophysiological role of stress hormones during development.

## Methods

### AAV constructs

Recombinant hAAT promoter-driven mouse GRα or green fluorescent protein (GFP) AAV vectors were constructed utilizing standard cloning methods with the coding sequence of mouse *NR3C1*. Vector DNA was packaged into AAV9 particles by use of triple-plasmid transfection of HEK293 cells^43,65^. Plasmids were purified by polyethylene glycol precipitation followed by CsCl centrifugation^66^. The titers of the purified viral stocks were determined by DNA dot blots as viral genomes (vg) per milliliter.

### Animal experiments

Female C57BL/6J mice randomized for experiments and male C57BL/6J mice were used as breeders for experiments (Jackson Laboratory; Bar Harbor, ME) and used between 12 and 14 weeks of age. All experiments of mice had ad libitum access to standard mouse chow and drinking water. Mice were exposed to a 12:12-h light/dark cycle. For pregnancy studies, male and female mice were set up for timed mating and the presence of a vaginal plug was marked at 0.5 dpc. Male mice were removed from the cage at 0.5 dpc and female mice were euthanized at either 14.5 dpc or PND1. Female mice not pregnant at 14.5 dpc were removed from the study. Glucocorticoid sensitivity was assessed in the livers of female mice at 14.5 dpc via injection of dexamethasone (10 μg/kg; I.P.) (Steraloids; Newport, RI). Four hours post I.P. injection, mice were killed and the livers were harvested. To reinstall GR during pregnancy, plugged mice were injected with 1 × 10^11^vg in 25 μl via retro-orbital venous sinus injection. All animal experiments were performed in at least two independent cohorts of animals to ensure reproducibility. All animal experiments were approved and performed in accordance with the Institutional Animal Care and Use Committee at the National Institute of Environmental Health Sciences, NIH.

### Transcriptome array

RNA was harvested from the livers of female virgins and mice pregnant at 14.5 dpc and used for whole-transcriptome analysis via Affymetrix GeneChip^®^ Mouse Exon 1.0ST Array (Thermo Fisher Scientific; Kalamazoo, MI). Briefly, 100 ng  of the total RNA was amplified and labeled with the Affymetrix WT Plus Reagent Kit. Five-and-a-half micrograms of amplified biotin–cDNAs were fragmented and hybridized to each array for 16 h at 45 °C in a rotating hybridization oven. Array slides were stained with streptavidin/PE utilizing a double antibody staining procedure, and washed with the Wash and Stain kit as per the manufacturer’s protocol FS450-0001. Slides were scanned in an Affymetrix Scanner 3000 and data were obtained using TAC software. The resulting data were processed using OmicSoft Array Studio (Qiagen; Redwood City, CA). Differentially expressed genes were detected with Partek Genomic Suite software and considered significantly different if they had *p* < 0.05 via ANOVA and passed a Benjamini–Hochberg multiple test correction for false positives with a *q* < 0.05. Heatmaps were generated with either R software package (version 3.3.3) or Partek Genomic Suite software.

### Quantitative PCR

One-hundred nanograms of the total RNA were reverse-transcribed and amplified according to the manufacturer’s instructions for the iScript One-Step RT-PCR kit for probes (Biorad; Hercules, CA). Quantitative real-time PCR (qPCR) was performed with the Biorad CFX96 sequence detection system using commercially available primer/probe sets (Applied Biosystems; Foster City, CA). Nascent *NR3C1* RNA was measured with primer/probes spanning either intron 1 (forward: 5′-GCAAATAACTCAGTACAAATGGTCT-3′; reverse: 5′-ACTATTTAACCAAACACCCAAAGG-3′) or the exon 2/intron 2 junction (forward: 5′-CGTGTGGAAGCTGTAAAGTC-3′; reverse: 5′-TGCACCTGAACTAATGTCTATCA-3′). The relative fluorescent signal of the gene of interest was normalized to PPIB using the 2^−ΔΔCT^ method.

### Immunoblotting

Glucocorticoid receptor protein expression was detected in liver lysates homogenized in SDS-sample buffer (Biorad) containing β-mercaptoethanol (Sigma Aldrich, St. Louis, MO). Thirty micrograms of protein were separated on 4–20% TGX gel (Biorad) and transferred onto a 0.2 μM nitrocellulose membrane (Biorad). Membranes were blocked with Licor blocking buffer (Licor, Lincoln, NE) followed by primary antibody incubation with anti-monoclonal glucocorticoid receptor antibody (1:1000) (Cell Signaling Technology, Beverly, MA) or anti-β-actin (1:5000) (Millipore, Bedford, MA). Protein was detected with fluorescent secondary antibody detection (1:10,000) (Licor) and imaged on the Licor Odyssey (Licor). Densitometry was measured with the Odyssey software, and protein loading was normalized with β-actin. Uncropped images of western blots presented in the article can be found in Supplementary Figure 3.

### Nanostring analysis

To measure mRNA expression, we performed Nanostring analysis from livers of virgins and 14.5 dpc pregnant mice injected with either AAV-GFP or AAV-GR using the PanCancer Pathway codeset (Nanostring Technologies, Seattle, WA) as previously described^67^. Briefly, 50 ng of the total RNA was hybridized at 65 °C for 20 h, and absolute RNA counts were measured with the nCounter as per the manufacturer’s protocol (Nanostring Technologies). Data were normalized to spiked-in positive and negative controls. Data were further normalized to *GAPDH* and reported as fold change over virgin (±SEM).

### Mitotic index

Two observers blindly counted the total observed mitotic figures in 20-x fields of hematoxylin- and eosin-stained slides. Counts were made at a multi-head microscope, and a determination of mitosis was made only if there was concordance between the observers. A mitotic index was calculated by taking the average mitotic number in 20 fields.

### Ki67 staining

Proliferating hepatocytes were confirmed with immunofluorescent staining of Ki67. Briefly, 4 μM frozen liver sections from 14.5 dpc pregnant AAV-GFP- and AAV-GR-injected mice were incubated with anti-Ki67 (1:200) (Abcam) overnight at 4 °C. Ki67+ staining was detected with Alexa-Fluor 594 secondary antibody (1:400) (Invitrogen, Carlsbad, CA). Nuclei were counterstained with DAPI, and Ki67+ nuclei were visualized with the Zeiss AxioD Observer Epifluorescent microscope (Zeiss Inc., Thornwood, NY). Ki67+ nuclei were quantified by counting the number of Ki67+ nuclei per field.

### Corticosterone measurements

Circulating maternal corticosterone from maternal blood samples was collected via submandibular bleed at ZT3 colorimetric corticosterone EIA according to the manufacturer’s instructions (Arbor Assays, Ann Arbor, MI). Whole embryos were homogenized, and the supernatants were used to measure fetal corticosterone with a colorimetric corticosterone EIA kit (Arbor Assays).

### Statistical analysis

Statistical significance was determined by using a two-sided Student’s *T* test when comparing two groups (Prism 7, Graphpad, La Jolla, CA, USA). A normality test was performed to determine distribution, and when failing, a Mann–Whitney post hoc test was performed. When comparing three or more groups, a two-way ANOVA was performed, followed by a Sidak post hoc test for multiple comparisons. Statistical significance was defined as *p* < 0.05 unless otherwise stated. Data are mean ± SEM (Prism 7).

### Reporting summary

Further information on experimental design is available in the Nature Research Reporting Summary linked to this article.

## Supplementary information

Reporting Summary

Supplementary Information

## Data Availability

Microarray data that support the findings of this study have been deposited in Gene Expression Omnibus (GSE121202). RNA-sequencing data used have been previously published^41^ and available on Gene Expression Omnibus (GSE99309). All other data supporting the conclusions of this paper are available from the corresponding author upon reasonable request.
