# Supplementary material for: Silencing of maternal hepatic glucocorticoid receptor is essential for normal fetal development in mice
Source: Commun Biol. 2019 Mar 15;2:104. doi: 10.1038/s42003-019-0344-3 (PMC6420645; doi:10.1038/s42003-019-0344-3)
Supplement: Supplementary file 1 — Reporting Summary [file 42003_2019_344_MOESM1_ESM.pdf]

nature research

Corresponding author(s): DBPR; COMMSBIO-18-1015A

Reporting Summary

Nature Research wishes to improve the reproducibility of the work that we publish. This form provides structure for consistency and transparency in reporting. For further information on Nature Research policies, see Authors & Referees and the Editorial Policy Checklist.

Statistical parameters

When statistical analyses are reported, confirm that the following items are present in the relevant location (e.g. figure legend, table legend, main text, or Methods section).

n/a	Confirmed	
☐	☒	The exact sample size (*n*) for each experimental group/condition, given as a discrete number and unit of measurement
☐	☐	An indication of whether measurements were taken from distinct samples or whether the same sample was measured repeatedly
☐	☒	The statistical test(s) used AND whether they are one- or two-sided<br>*Only common tests should be described solely by name; describe more complex techniques in the Methods section.*
☒	☐	A description of all covariates tested
☐	☒	A description of any assumptions or corrections, such as tests of normality and adjustment for multiple comparisons
☐	☒	A full description of the statistics including central tendency (e.g. means) or other basic estimates (e.g. regression coefficient) AND variation (e.g. standard deviation) or associated estimates of uncertainty (e.g. confidence intervals)
☐	☒	For null hypothesis testing, the test statistic (e.g. *F*, *t*, *r*) with confidence intervals, effect sizes, degrees of freedom and *P* value noted<br>*Give P values as exact values whenever suitable.*
☒	☐	For Bayesian analysis, information on the choice of priors and Markov chain Monte Carlo settings
☒	☐	For hierarchical and complex designs, identification of the appropriate level for tests and full reporting of outcomes
☒	☐	Estimates of effect sizes (e.g. Cohen's *d*, Pearson's *r*), indicating how they were calculated
☐	☒	Clearly defined error bars<br>*State explicitly what error bars represent (e.g. SD, SE, CI)*

Our web collection on statistics for biologists may be useful.

Software and code

Policy information about availability of computer code

Data collection	Arrays were scanned in an Affymetrix Scanner 3000 and data was obtained using TAC Software. Nanostring data was collected with nCounter Digital Analyzer.
Data analysis	Whole transcriptome microarray data was analyzed with OmicSoft Array Studio (Version 10.1) software. Nanostring data was analyzed with nSolver (Version 3.0).Unpaired T-Test was

For manuscripts utilizing custom algorithms or software that are central to the research but not yet described in published literature, software must be made available to editors/reviewers upon request. We strongly encourage code deposition in a community repository (e.g. GitHub). See the Nature Research guidelines for submitting code & software for further information.

Data

Policy information about availability of data

All manuscripts must include a data availability statement. This statement should provide the following information, where applicable:

- Accession codes, unique identifiers, or web links for publicly available datasets
- A list of figures that have associated raw data
- A description of any restrictions on data availability

Microarray data that support the findings of this study have been deposited in Gene Expression Omnibus (GSE121202). RNA-sequencing data used has been previously published and available on Gene Expression Omnibus (GSE99309)

Field-specific reporting

Please select the best fit for your research. If you are not sure, read the appropriate sections before making your selection.

☒ Life sciences ☐ Behavioural & social sciences ☐ Ecological, evolutionary & environmental sciences

For a reference copy of the document with all sections, see nature.com/authors/policies/ReportingSummary-flat.pdf

Life sciences study design

All studies must disclose on these points even when the disclosure is negative.

Sample size	No methods were used to determine sample size. Initial experiments examining GR protein expression (Fig. 1E) utilized 9 mice. Results were robust and repeatable. In our experience, immunoblotting data historically needed to be powered more than gene expression and ChIP analyses. Therefore, in an effort to confer to the 3R's of in vivo animal research we performed follow up gene expression experiments in cohorts of 3 mice per group.
Data exclusions	Two mice were removed from the study for maternal weight gain experiments as at time of sacrifice mice were not pregnant.
Replication	Different cohorts of mice were used for follow up experiments. Each figure contains a separate cohort of mice to ensure reproducibility of our results. AAV experiments in pregnant dams was performed over 3 separate cohorts to ensure reproducibility of injections and of our biological findings.
Randomization	Mice were randomly assigned into groups (virgins vs. pregnant; AAV-GFP vs. AAV-GR).
Blinding	Histological examination of AAV-injected pregnant mouse livers was examined in a blinded fashion.

Reporting for specific materials, systems and methods

Materials & experimental systems

n/a	Involved in the study
☒	☐ Unique biological materials
☐	☒ Antibodies
☒	☐ Eukaryotic cell lines
☒	☐ Palaeontology
☐	☒ Animals and other organisms
☒	☐ Human research participants

Methods

n/a	Involved in the study
☒	☐ ChIP-seq
☒	☐ Flow cytometry
☒	☐ MRI-based neuroimaging

Antibodies

Antibodies used	The glucocorticoid receptor antibody was purchased from Cell Signaling Technology (cat #3660, clone D8H2). ChIP grade YY1 antibody was purchased from Abcam (cat#ab38422).
Validation	GR antibody specificity was confirmed utilizing tissue specific GR knockout animals generated in our lab (Quinn & Cidlowski, FASEB J 2016). YY1 antibody used for ChIP was performed according to manufacturer's suggested dilution.

April 2018

Animals and other organisms

Policy information about studies involving animals; ARRIVE guidelines recommended for reporting animal research

Laboratory animals	Female and male C57BL6 mice were used in all studies as described in the material and methods. Animal husbandry is explained in the materials and methods.
Wild animals	Study did not involve wild animals.
Field-collected samples	Study did not involve samples collected in the field.
