# Supplementary material for: Silencing of maternal hepatic glucocorticoid receptor is essential for normal fetal development in mice
Source: Commun Biol. 2019 Mar 15;2:104. doi: 10.1038/s42003-019-0344-3 (PMC6420645; doi:10.1038/s42003-019-0344-3)
Supplement: Supplementary file 2 — Supplementary Information [file 42003_2019_344_MOESM2_ESM.pdf]

Supplemental Figure 1

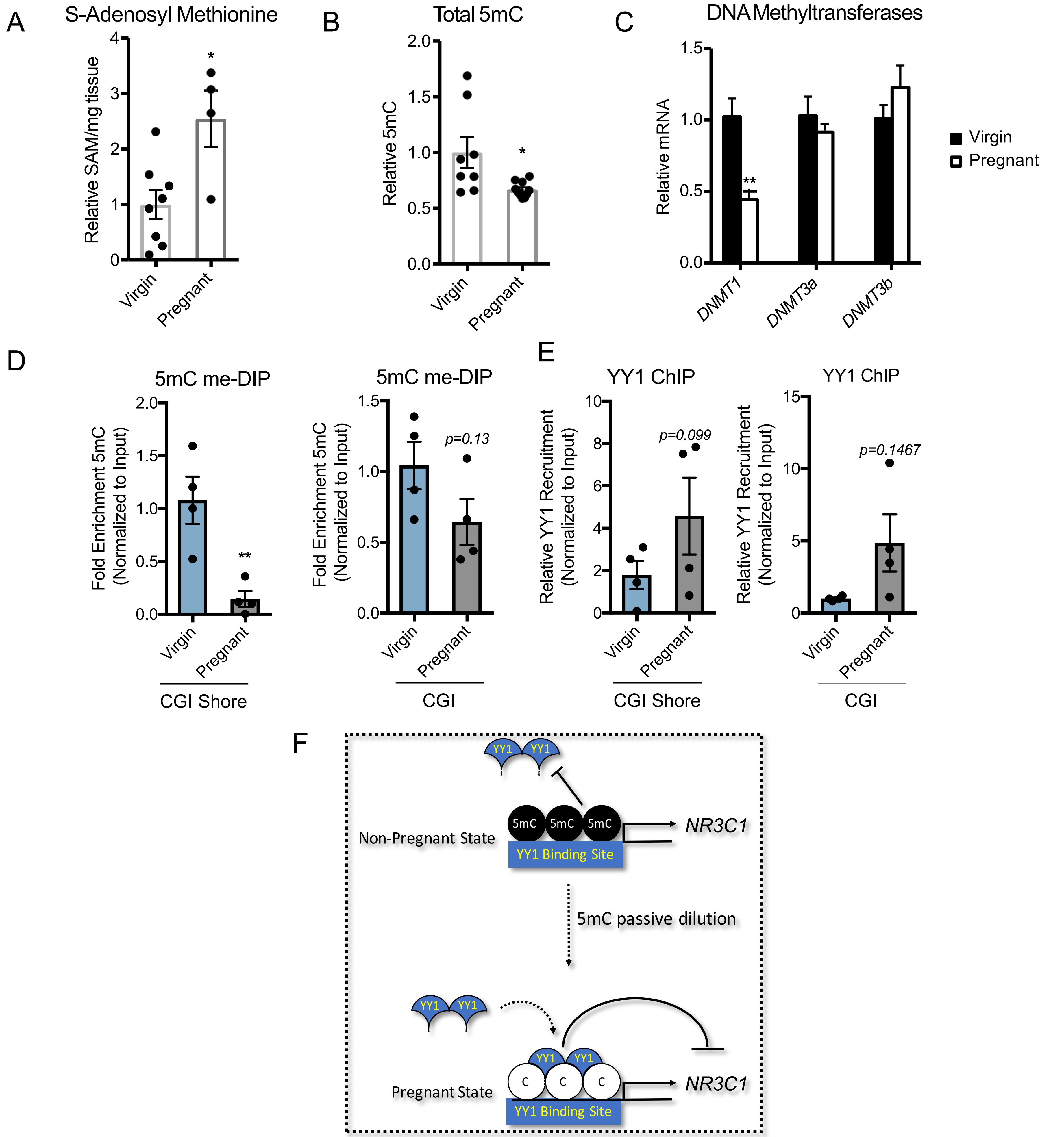

Supplemental Figure 1: Epigenetic regulation of maternal hepatic GR during pregnancy. (A) Relative S- adenosyl methionine levels in livers from virgin and 14.5dpc pregnant mice. Data are expressed as relative SAM levels/mg tissue compared to virgin mice.. n=4-8 mice per group. (B) Relative 5mC levels in livers from virgin and 14.5dpc pregnant mice. Data are expressed as relative 5mC compared to virgins ± SEM. n=8-10 mice per group. (C) Relative mRNA expression of *DNMT1, DNMT3a and DNMT3b* assessed by qPCR in livers from virgin and 14.5dpc pregnant mice. (D) 5mC-meDIP of NR3C1 CGI Shore and CGI in virgin and pregnant liver. n=4 mice per group. (E) ChIP of YY1 to NR3C1 CGI Shore and CGI in virgin and pregnant liver. n=4 mice per group. (F) Schematic representation of YY1-mediated GR repression during pregnancy. Data are expressed as relative expression of virgins ± SEM. n=4-6 mice per group. * denotes $p<0.05$, ** $p<0.01$.

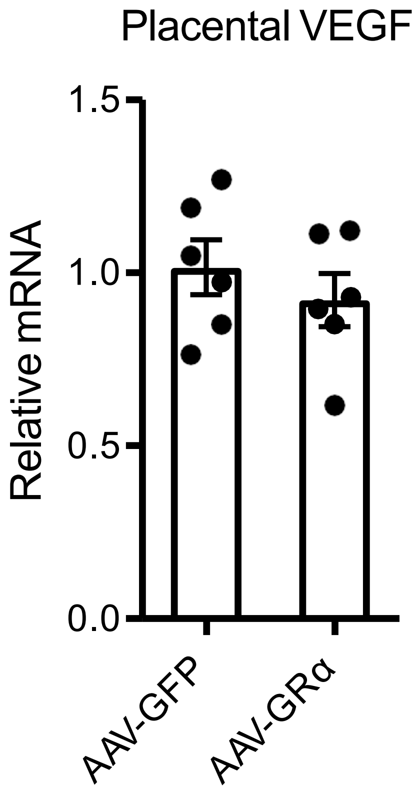

Supplemental Figure 2: Hepatic GR reinstallation does not alter placental VEGF expression. Quantitative real-time PCR of VEGF mRNA expression in placentas taken from AAV-GFP and AAV-GR injected mice at 14.5dpc. n=6 placentas per group.

Supplemental Figure 3

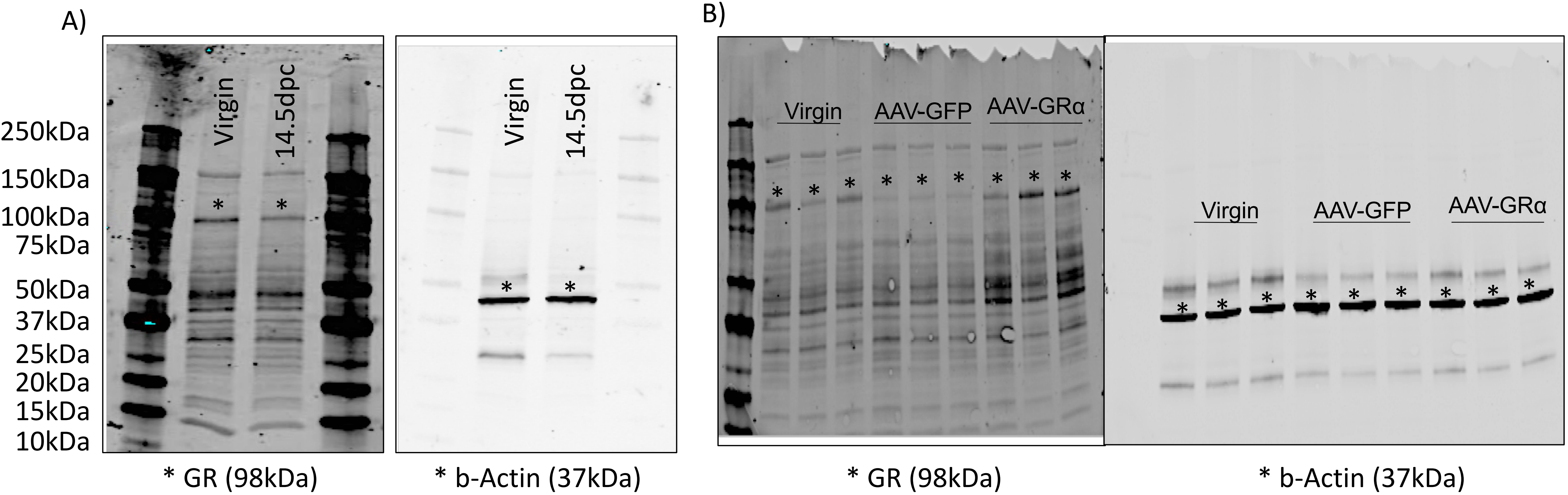

Supplemental Figure 3: Uncropped western blot images from manuscript. (A) Western blot image from figure 1e uncropped. Left image is for GR and right image is for b-actin. (B) Western blot image from figure 2a uncropped. Left image is for GR and right image is for b-actin. * denotes band of interest for quantification in main text.

Supplemental Methods

S-adnosylmethionine measurements

Liver tissue from virgin and 14.5dpc pregnant females was excised and 1 gram of tissue was homogenized in 5ml ice cold PBS with a Dounce homogenizer for 15 strokes. Lysates were centrifuged for 15 minutes at 4ºC at 10,000g and supernatants collected. S-adnosylmethionine was assayed with the S-adenosylmethionine ELISA kit according to manufacturer's protocol (Cell Biolabs Inc, San Diego, CA, USA).

Total 5mC measurement

Total genomic DNA was isolated from livers of virgin and 14.5dpc pregnant mice using the Qiagen DNeasy blood and tissue kit according to manufacturer's protocol. Total 5mC was detected
in isolated genomic DNA using the MethylFlash Methylated DNA quantification kit (Epigentek, Farmingdale, NY, USA) following the manufacturer's protocol.

5mC-meDIP

Genomic DNA was isolated from livers of virgin and 14.5dpc pregnant mice and sheared via sonication (3 cycles, 15 seconds on 30 seconds off). Three micrograms of sheared DNA was immunoprecipitated and purified using the commercially available Methylamp□ Methylated DNA Capture Kit (Epigentek; Farmingdale, NY) according to manufacturer's protocol. 5mC enriched DNA fragments were used as templates to assess 5mC deposition at the mouse NR3C1 CGI and CGI shore via qPCR.

Chromatin immunoprecipitation

Approximately 100mg of liver tissue from virgin and 14.5dpc pregnant mice was cross-linked and nuclei isolated using the EZ-Magna ChIP kit according to manufacturer's protocol (Millipore). Isolated DNA was sonicated (15 cycles high; 30 seconds on, 30 seconds off) (Diagenode bioruptor; Denville, NJ). Sheared DNA was immunoprecipitated with anti-YY1 antibody (10µg) (Abcam; Cambridge, MA) overnight followed by pull down using the magnetic beads. Immunoprecipitated DNA was purified via the QIAquick PCR purification kit (Qiagen) and eluted in 50µl of elution buffer. The following primers were used for quantitative analysis of the CpG island shores (CGI shore) of mouse *NR3C1*: Forward: 5' TCCTTCTTGAGGTGTCAAGCTTC 3'; Reverse: 5' ATGTAAAGGCTGCCCAATGTG 3' and mouse CGI; Forward: 5' CTTGGCACTTCTGATCGGAG 3'; Reverse: 5' AATGGTGACCGTGTGGCGTC 3'.
